# Supplementary material for: A Biological-Systems-Based Analyses Using Proteomic and Metabolic Network Inference Reveals Mechanistic Insights into Hepatic Lipid Accumulation: An IMI-DIRECT Study
Source: medRxiv. 2025 Jun 2:2025.06.02.25328773. Preprint. [Version 1] doi: 10.1101/2025.06.02.25328773 (PMC12155020; doi:10.1101/2025.06.02.25328773)
Supplement: Supplement 1 [file NIHPP2025.06.02.25328773v1-supplement-1.pdf]

medRxiv preprint doi: https://doi.org/10.1101/2025.06.02.25328773; this version posted June 2, 2025. The copyright holder for this preprint (which was not certified by peer review) is the author/funder, who has granted medRxiv a license to display the preprint in perpetuity.
It is made available under a CC-BY 4.0 International license .

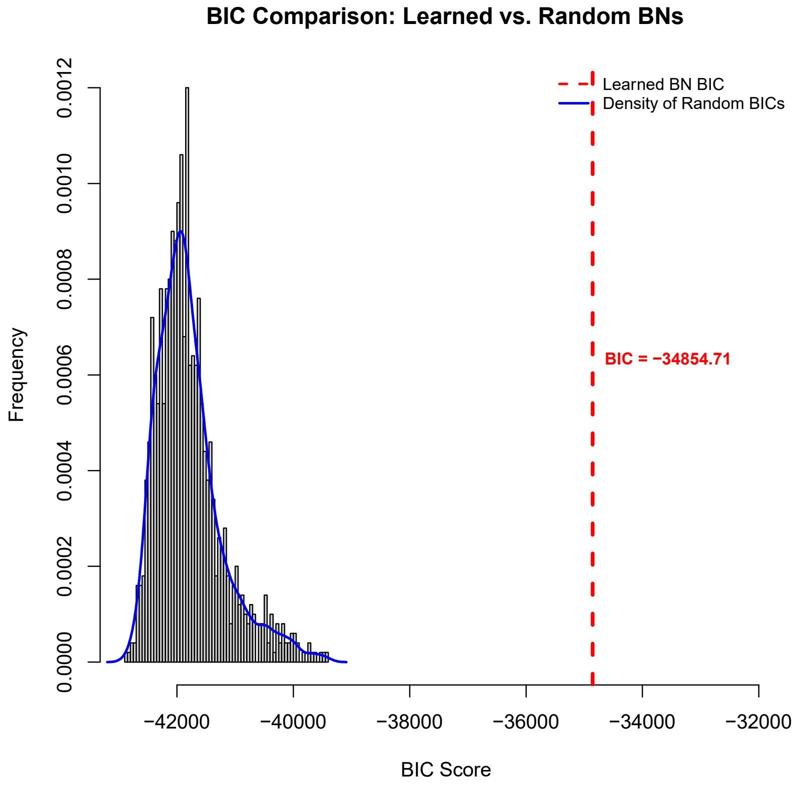

Figure S1. Bayesian Information Criterion (BIC) comparison for non-diabetes network model validation (n=964)

Histogram of BIC scores from 1,000 randomly generated null networks (gray bars) with a smoothed density overlay (blue line). The red dashed line shows the BIC of the learned network (BIC = -34854.71), indicating significantly better fit than expected by chance.

medRxiv preprint doi: https://doi.org/10.1101/2025.06.02.25328773; this version posted June 2, 2025. The copyright holder for this preprint (which was not certified by peer review) is the author/funder, who has granted medRxiv a license to display the preprint in perpetuity.
It is made available under a CC-BY 4.0 International license .

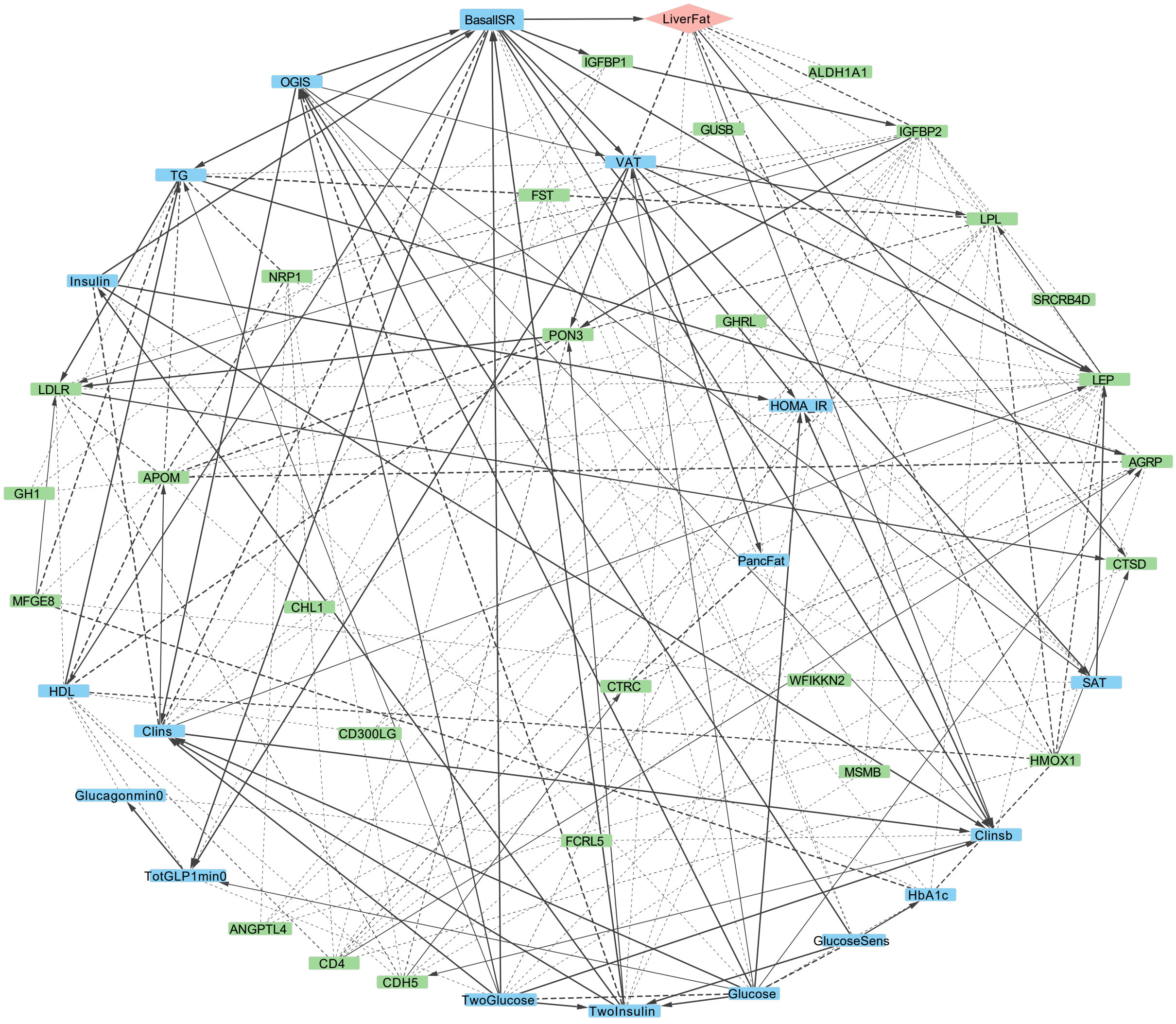

Figure S2. Expanded Bayesian network of metabolic and proteomic interactions in the IMI-DIRECT non-diabetes cohort (n = 964).

This detailed graph shows all directed relationships (arcs with strength ≥ 0.5) among clinical and proteomic variables. Line thickness reflects the strength of association. Nodes are color-coded as follows: blue for clinical/metabolic variables, green for proteins, and peach for liver fat (outcome). Solid arrows represent directed associations with high confidence (strength and directional probability ≥ 0.8), while dashed arrows indicate less confident directionality.

AGRP: agouti-related peptide; ALDH1A1: aldehyde dehydrogenase 1 family member A1; APOM: apolipoprotein M; BasalISR: basal insulin secretion rate at the beginning of the OGTT; CD4: cluster of differentiation 4; CDH5: cadherin-5; Clins: mean insulin clearance during OGTT; Clinsb: basal insulin clearance-calculated as (mean insulin secretion)/(mean insulin concentration); CTRC: chymotrypsin C; CTSD: cathepsin D; FGF21: fibroblast growth factor 21; Glucagonmin0: fasting glucagon; Glucose: fasting plasma glucose; GlucoseSens: glucose sensitivity; GUSB: β-glucuronidase; HbA1c: glycated haemoglobin A1C; HDL: high-density lipoprotein cholesterol; IGFBP1/2: insulin-like growth factor binding proteins 1 and 2; Insulin: fasting plasma insulin; KITLG: KIT ligand; LDLR: low-density lipoprotein receptor; LEP: leptin; LiverFat: hepatic fat content; LPL: lipoprotein lipase; MFGE8: milk fat globule-epidermal growth factor 8; OGIS: oral glucose insulin sensitivity index according to the method of Mari et al. [32]; PancFat: pancreas fat; PON3: paraoxonase 3; SAT: subcutaneous adipose tissue; TG: triglycerides; TotGLP1min0: fasting total GLP-1; TwoGlucose/TwoInsulin: 2-hour post-load values from OGTT; VAT: visceral adipose tissue.

medRxiv preprint doi: https://doi.org/10.1101/2025.06.02.25328773; this version posted June 2, 2025. The copyright holder for this preprint (which was not certified by peer review) is the author/funder, who has granted medRxiv a license to display the preprint in perpetuity.
It is made available under a CC-BY 4.0 International license .

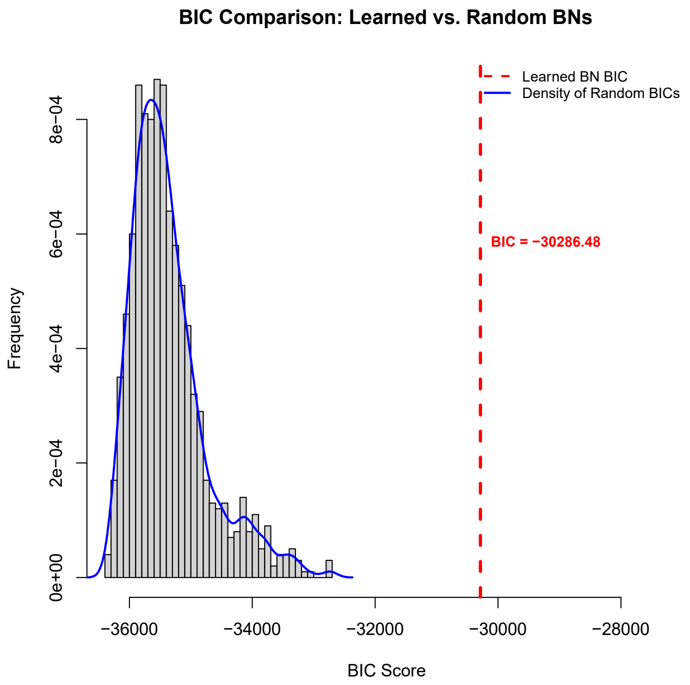

Figure S3. Bayesian Information Criterion (BIC) comparison for diabetes network model validation (n=331)

Histogram of BIC scores from 1,000 randomly generated null networks (gray bars) with a smoothed density overlay (blue line). The red dashed line shows the BIC of the learned network (BIC = -30286.48), indicating significantly better fit than expected by chance.

medRxiv preprint doi: https://doi.org/10.1101/2025.06.02.25328773; this version posted June 2, 2025. The copyright holder for this preprint (which was not certified by peer review) is the author/funder, who has granted medRxiv a license to display the preprint in perpetuity.
It is made available under a CC-BY 4.0 International license .

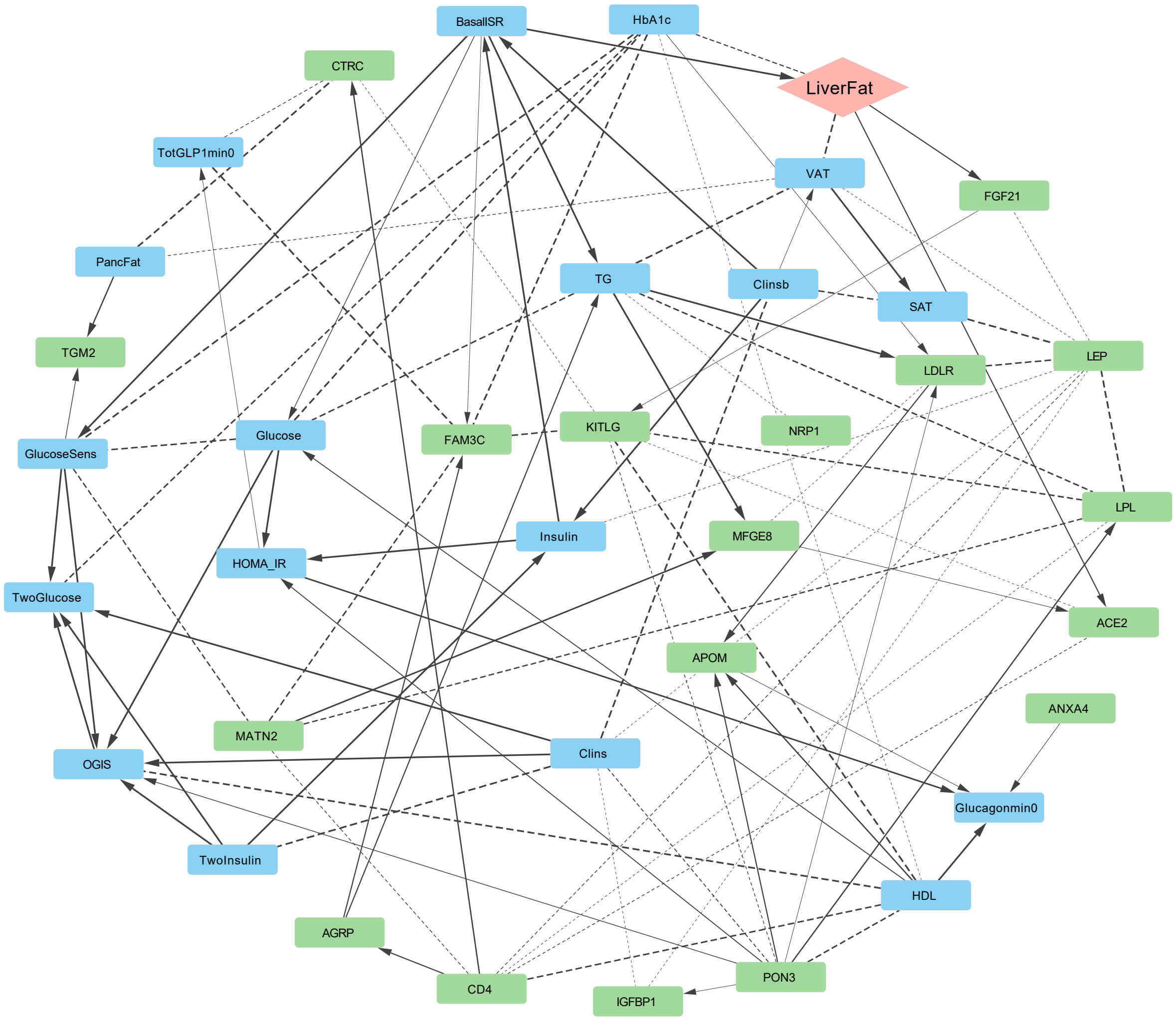

Figure S4. Expanded Bayesian network of metabolic and proteomic interactions in the IMI-type 2 diabetes cohort (n = 331).

This detailed graph shows all directed relationships (arcs with strength ≥ 0.5) among clinical and proteomic variables. Line thickness reflects the strength of association. Nodes are color-coded as follows: blue for clinical/metabolic variables, green for proteins, and peach for liver fat (outcome). Solid arrows represent directed associations with high confidence (strength and directional probability ≥ 0.8), while dashed arrows indicate less confident directionality.

ACE2: angiotensin-converting enzyme 2; AGRP: agouti-related peptide; ALDH1A1: aldehyde dehydrogenase 1 family member A1; ANXA4: annexin A4; APOM: apolipoprotein M; BasalISR: basal insulin secretion rate; CD4: cluster of differentiation 4; Clins: mean insulin clearance during OGTT; Clinsb: basal insulin clearance; CTRC: chymotrypsin C; FGF21: fibroblast growth factor 21; FAM3C: family with sequence similarity 3 member C; Glucagonmin0: fasting glucagon; Glucose: fasting plasma glucose; GlucoseSens: glucose sensitivity; HDL: high-density lipoprotein cholesterol; HOMA_IR: homeostatic model assessment of insulin resistance; IGFBP1: insulin-like growth factor binding protein 1; Insulin: fasting plasma insulin; KITLG: KIT ligand; LDLR: low-density lipoprotein receptor; LEP: leptin; LiverFat: hepatic fat content; LPL: lipoprotein lipase; MATN2: matrilin-2; MFGE8: milk fat globule-EGF factor 8; NRP1: neuropilin-1; OGIS: oral glucose insulin sensitivity index according to the method of Mari et al. [32]; PancFat: pancreas fat; PON3: paraoxonase 3; SAT: subcutaneous adipose tissue; TG: triglycerides; TGM2: transglutaminase 2; TotGLP1min0: fasting total GLP-1; TwoGlucose: 2-hour post-load glucose (OGTT); TwoInsulin: 2-hour post-load insulin (OGTT); VAT: visceral adipose tissue.

medRxiv preprint doi: https://doi.org/10.1101/2025.06.02.25328773; this version posted June 2, 2025. The copyright holder for this preprint
(which was not certified by peer review) is the author/funder, who has granted medRxiv a license to display the preprint in perpetuity.
It is made available under a CC-BY 4.0 International license .

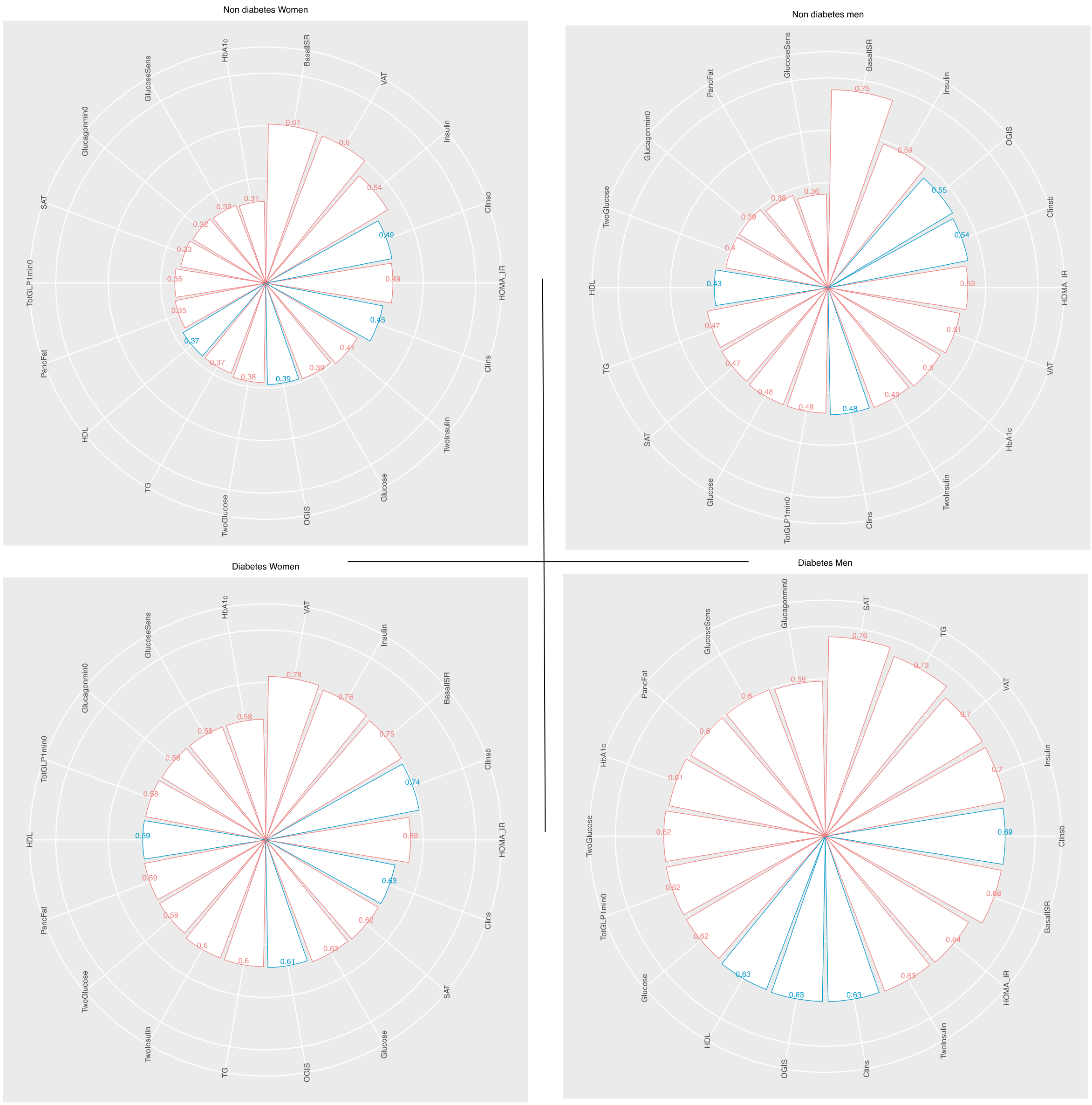

Figure S5. Sex-stratified Posterior probabilities of MASLD based on clinical predictors across IMI-DIRECT diabetes and non-diabetes cohorts.

Radar plots display MASLD conditional probabilities following conditioning on individual clinical variables, stratified by sex and diabetes status: non-diabetic females, n=171 (top left), non-diabetic males, n=793 (top right), diabetic females, n=139 (bottom left), and diabetic males, n=193 (bottom right). Red bars indicate high levels and blue bars low levels of the respective variable.

MASLD: metabolic dysfunction-associated steatotic liver disease; T2D: type 2 diabetes; BasalISR: basal insulin secretion rate; VAT: visceral adipose tissue; SAT: subcutaneous adipose tissue; OGIS: oral glucose insulin sensitivity index; HOMA_IR: homeostatic model assessment of insulin resistance; Clinsb: basal insulin clearance; Clins: dynamic insulin clearance; TG: triglycerides; HDL: high-density lipoprotein cholesterol; HbA1c: glycated haemoglobin A1c; GLP1: glucagon-like peptide 1; OGTT: oral glucose tolerance test.

medRxiv preprint doi: https://doi.org/10.1101/2025.06.02.25328773; this version posted June 2, 2025. The copyright holder for this preprint (which was not certified by peer review) is the author/funder, who has granted medRxiv a license to display the preprint in perpetuity.
It is made available under a CC-BY 4.0 International license .

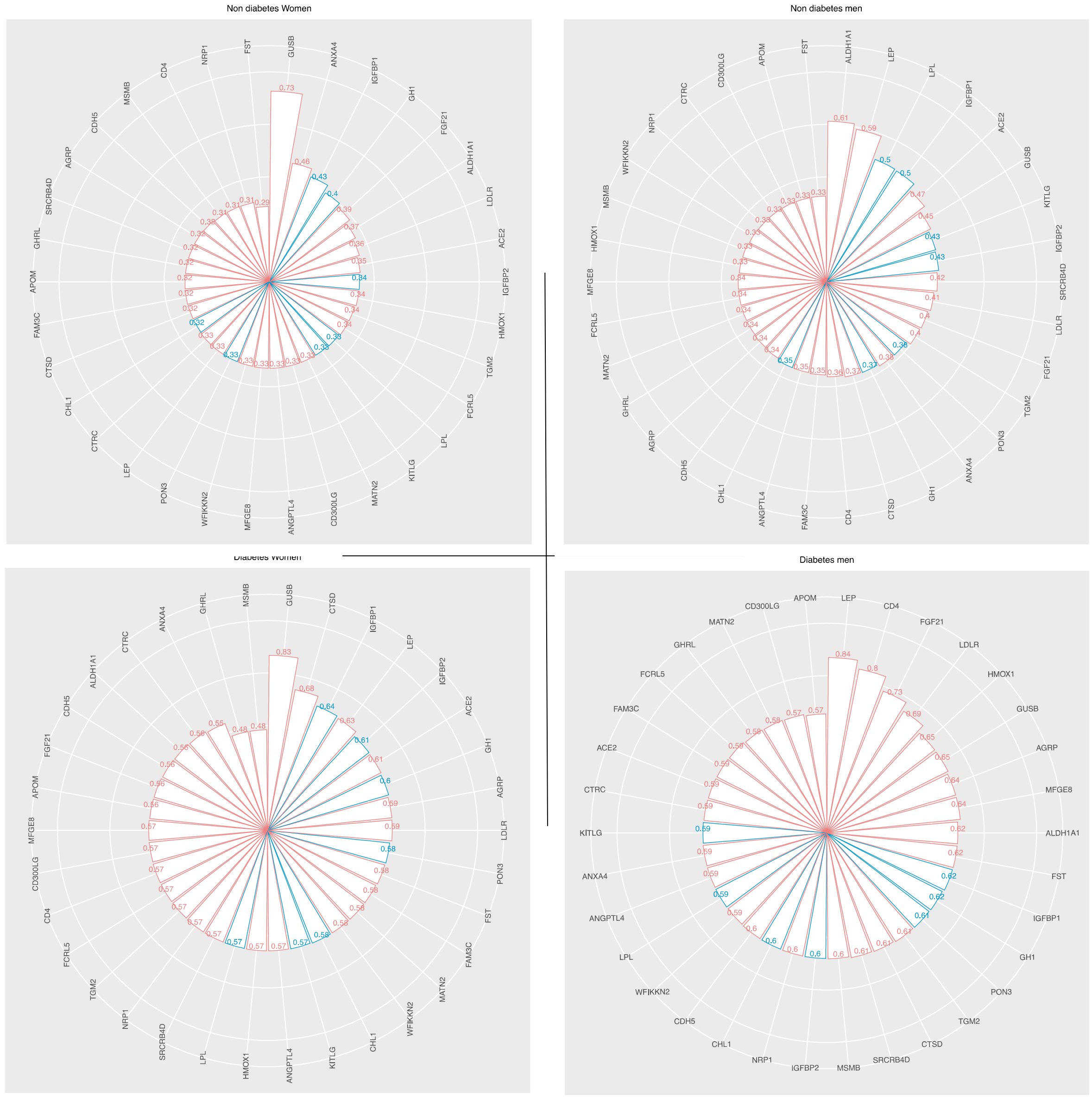

Figure S6. Sex-stratified Posterior probabilities of MASLD based on proteomic predictors in IMI-DIRECT diabetes and non-diabetes cohorts.

Radar plots display MASLD conditional probabilities after conditioning on proteomic variables, stratified by sex and diabetes status: non-diabetic females, n=171 (top left), non-diabetic males, n=793 (top right), diabetic females, n=138 (bottom left), and diabetic males, n=193 (bottom right). Red bars show the conditional probability given high levels, and blue bars indicate conditioning on low levels of each protein.

MASLD: metabolic dysfunction-associated steatotic liver disease; T2D: type 2 diabetes; GUSB: β-glucuronidase; LEP: leptin; ALDH1A1: aldehyde dehydrogenase 1 family member A1; CTSD: cathepsin D; FGF21: fibroblast growth factor 21; IGFBP1/2: insulin-like growth factor binding proteins 1 and 2; LDLR: low-density lipoprotein receptor; MFGE8: milk fat globule-EGF factor 8; AGRP: agouti-related peptide; KITLG: KIT ligand; ACE2: angiotensin-converting enzyme 2; APOM: apolipoprotein M; CD4: cluster of differentiation 4; PON3: paraoxonase 3; TGM2: transglutaminase 2; MATN2: matrilin-2.
